# VMAT for the treatment of gynecologic malignancies for patients unable to receive HDR brachytherapy

**DOI:** 10.1120/jacmp.v15i5.4839

**Published:** 2014-09-08

**Authors:** Caitlin Merrow, Steven deBoer, Matthew B. Podgorsak

**Affiliations:** ^1^ Department of Radiation Medicine Roswell Park Cancer Institute Buffalo NY USA

**Keywords:** volumetric‐modulated arc therapy (VMAT), stereotactic body radiation therapy (SBRT), cervical cancer, brachytherapy

## Abstract

This investigation studies the use of volumetric‐modulated arc therapy (VMAT) to deliver the following conceptual gynecological brachytherapy (BT) dose distributions: Type 1, traditional pear‐shaped dose distribution with substantial dose gradients; Type 2, homogeneous dose distribution throughout PTV (BT prescription volume); and Type 3, increased dose to PTV without organ‐at‐risk (OAR) overdose. A tandem and ovoid BT treatment plan, with the prescription dose of 6 Gy to point A, was exported into the VMAT treatment planning system (TPS) and became the baseline for comparative analysis. The 200%, 150%, 130%, 100%, 75%, and 50% dose volumes were converted into structures for optimization and evaluation purposes. The 100% dose volume was chosen to be the PTV. Five VMAT plans (Type 1) were created to duplicate the Ir‐192 tandem and ovoid inhomogeneous dose distribution. Another five VMAT plans (Type 2) were generated to deliver a homogeneous dose of 6 Gy to the PTV. An additional five VMAT plans (Type 3) were created to increase the dose to the PTV with a homogeneous dose distribution. In the first set of plans, the dose given to 99% of the 200%–100% dose volumes agreed within 2% of the BT plan on average. Additionally, it was found that the 75% dose volumes agreed within 5% of the BT plan and the 50% dose volumes agreed within 6.4% of the BT plan. In the second set of comparative analyses, the 100% dose volume was found to be within 1% of the original plan. Furthermore, the maximum increase of dose to the PTV in the last set of comparative analyses was 8 Gy with similar doses to OARs as the other VMAT plans. The maximum increase of dose was 2.50 Gy to the rectum and the maximum decrease of dose was 0.70 Gy to the bladder. Henceforth, VMAT was successful at reproducing brachytherapy dose distributions demonstrating that alternative dose distributions have the potential to be used in lieu of brachytherapy. It should also be noted that differences in radiobiology need to be further investigated.

PACS numbers: 87.50.‐a, 87.53.‐j, 87.55.‐x, 87.55.D‐, 87.55.dk, 87.55.de

## I. INTRODUCTION

Brachytherapy is commonly used in radiation therapy for the treatment of cervical cancer. The development of remote after loader high‐dose‐rate (HDR) systems has made brachytherapy a more accessible and safer treatment option for patients and personnel alike. Tandem and ovoid treatment plans using HDR brachytherapy provide a unique pear‐shaped dose distribution with steep dose gradients. However, they also contain certain limitations concerning patient specific source configurations, cause more patient discomfort, and are subject to errors due to applicator movement or alignment between fractions, insertion of the device, and image acquisition.^(1)^

Patients who do not receive surgical intervention for their disease are commonly treated with conventional external beam radiation therapy (EBRT) with a prescription dose of 45 Gy in 25 fractions, followed by a brachytherapy boost to the primary tumor of 6 Gy to point A per fraction for 5 fractions, resulting in a total dose of 75 Gy.^(2)^ Despite the improvements in treating cervical cancer with HDR, there are patients who are unable or unwilling to receive brachytherapy treatment for their disease. In these situations alternative therapy must be used in an attempt to control the disease.

When a patient is unable to receive brachytherapy as a treatment modality, external beam radiation therapy is often used as an alternative. Typically an external beam stereotactic boost of 4–6 Gy for 3 to 5 fractions is administered, instead of the previously mentioned brachytherapy treatment.^3^, ^4^, ^5^, ^6^ The isodose lines of an HDR tandem and ovoid plan are significantly different than those of an external beam treatment plan and should be taken into consideration when deliberating the alternatives. Furthermore, the methods of evaluating the dose to the organs at risk (i.e., the bladder and rectum) in an HDR plan are different than those in an external beam plan. For instance, in an HDR treatment plan the dose to the rectum or bladder is often evaluated by the dose to the point on the organ nearest the source.^(7)^ This most adjacent point to the source in the organ is, by nature of the radioactive source, the maximum dose point in the organ. In external beam treatment plans, not only are maximum dose points to organs at risk considered, dose volumes are taken into account as well.^7^, ^8^ These differences in dose evaluation could lead to an unintended compromise in a patient's treatment if they are not addressed appropriately.

The purpose of this study is to demonstrate and dosimetrically evaluate the potential of VMAT for the treatment of cervical cancer with homogeneous and inhomogeneous dose distributions using the traditional dose regimen of 6 Gy to point A for five fractions, as well as nontraditional dose regimens of 7–10 Gy to point A using homogenous dose distributions.

## II. MATERIALS AND METHODS

### A. Treatment planning volumes

A single fraction of a tandem and ovoid treatment plan with the prescription dose of 6 Gy per fraction to point A for 5 fractions was chosen to be exported from Nucletron's Oncentra Brachy radiotherapy treatment planning system (TPS) (Nucletron BV, Veenendaal, The Netherlands) to Varian Medical Systems Inc., Eclipse version 10.0 (Varian Medical Systems, Palo Alto, CA). The same computed tomography images were used for both the tandem and ovoid treatment plan and the external beam plans. In Eclipse TPS, the body, femoral heads, bladder, and rectum were all contoured. Also in the Eclipse TPS, the 200%, 150%, 130%, 100%, 75%, and 50% isodose lines were converted into treatment planning structures for optimization and dose evaluation purposes. The 100% isodose volume was designated as the planning treatment volume (PTV). All dosimetric analysis was completed in the Eclipse TPS. The point A and point B dose points were also created in the Eclipse TPS. A dose‐volume histogram of the newly contoured and converted dose structures of the tandem and ovoid plan was then used as a baseline for evaluating the maximum dose points and dose volumes of the organs at risk and point A and B doses.

### B. Treatment planning

The brachytherapy treatment plan was planned in Nucletron's Oncentra brachytherapy TPS. All VMAT treatment plans were planned using Varian Medical Systems Inc., Eclipse version 10.0. The isocenter was chosen to correspond closely to the center of mass of the 100% isodose volume. No normalization points were chosen for the brachytherapy treatment plan or the VMAT treatment plans. Tissue density corrections were applied to all of the VMAT treatment plans. This was done to account for the differences in attenuation of the tandem and ovoid applicator and the surrounding tissues. The original brachytherapy plan did not utilize any tissue heterogeneity corrections. The brachytherapy treatment plan was planned using Ir‐192 source data with an average energy of about 0.38 MeV.^(9)^ All VMAT plans were planned using 6 MV photons.

Three of the Type 1 VMAT plans utilized one full arc, while the remaining two plans used two full noncoplanar arcs with a separation of 10° between arcs. One of the five Type 2 VMAT plans consisted of two full noncoplanar arcs with a separation of 10° between arcs. The remaining four Type 2 plans created to deliver the homogenous dose distribution used two noncoplanar partial arcs with a 5°–15° separation and were chosen with the intent of sparing organs at risk. Collimator angles of 45° and 315° were chosen in order to minimize the MLC tongue‐and‐groove effects and to provide the ability for higher modulation than a collimator angle of 0°. Two of the Type 3 VMAT plans utilized a mix of one to two full and/or partial noncoplanar arcs with a 5°–25° separation between arcs. The remaining three Type 3 plans consisted of three full noncoplanar arcs, each with a different isocenter. The isocenters of the three different arcs were chosen to be 1) in the center of the right ovoid, 2) in the center of the left ovoid, and 3) in the center of the tandem. The technique of using multiple isocenters for multiple arcs was used to aid in the sparing of the organs at risk and to increase dose coverage to the PTV margin.

Upper and lower objectives with priorities of 150 and 130, respectively, were also used for the 200%, 150%, 130%, 75%, and 50% dose volume structures for the five Type 1 VMAT plans. These upper and lower objective functions were set to correlate to the appropriate doses of each of the dose volume structures. No upper and lower objectives were placed on the 200%, 150%, 130%, 75%, and 50% dose volume structures for the Type 2 VMAT plans. These objectives were only placed on the PTV and PTV+1mm structures with upper and lower priorities of 150 and 130, respectively. The Type 3 VMAT plans consisted of an upper objective of 0.0% of the PTV structure receiving 110% of the Rx dose and a lower objective of 100.0% of the PTV structure receiving 100% of the Rx dose. The Type 3 VMAT plans consisted of upper and lower objectives similar to the previously mentioned, but were simply increased in dose before and during optimization.

The maximum dose limits and dose volume constraints to the OARs were first set to the RTOG 0417‐recommended dose limits and were then changed to more conservative maximum dose points and dose volume constraints once the first level of optimization was complete.^(10)^ The priorities of the OARs were initially set to 90 and then increased throughout the optimization levels to further decrease the dose to these critical structures without compromising the PTV coverage. The priorities of the upper objectives pertaining to the OARs were never allowed to exceed those of the PTV, PTV+1mm, and dose volume structures.

The organs at risk were all given a priority of 90 at the beginning of optimization of all 15 plans. A structure equal to the 6 Gy isodose volume was created and said to be the PTV. An additional structure equal to the PTV+1mm in all directions was created and used during optimization in order to aid in dose comformality and dose coverage to the PTV. Upper and lower objectives with priorities of 150 and 130, respectively, were used for the PTV and PTV+1mm for all of the VMAT plans.

#### C. Evaluation of treatment plans

The brachytherapy and VMAT treatment plans were evaluated by comparing target dose coverage, isodose volume coverage, and critical structure dose. The critical structures evaluated were the bladder, rectum, left femoral head, and right femoral head. The brachytherapy treatment plan exported into Eclipse TPS became the baseline for all comparative analysis. The 200%, 150%, 130%, 75%, and 50% dose volumes converted to dose volume structures were used during optimization and dose evaluation. The dose received by 99.0% of each dose structure for all dose volumes in the 15 VMAT treatment plans was compared to the dose received by 99.0% of each dose structure in the brachytherapy treatment plan.

Point A and point B dose specifications defined in the Manchester System were used during dose evaluation for all 15 VMAT treatment plans, as well as the original brachytherapy treatment plan.^9^, ^11^ These points were the same in both the brachytherapy and VMAT treatment plans. Point A is defined as the point located 2 cm superior to the cervical os and 2 cm lateral to the cervical canal. If the cervical canal is displaced, then point A should remain in the plane of the cervical canal. Point B is defined as the point located 3 cm lateral to point A. In the event that the cervical canal is displaced and point A is in plane with the cervical canal, point B is to remain 5 cm from the midline.^(11)^

Dose‐volume histograms were used in order to evaluate the maximum dose points and dose volumes of the organs at risk. It should be noted that, due to the increase of integral dose when using VMAT, the Manchester System of dose specification of the OARs was not appropriate for our purposes. The maximum dose points to the bladder, rectum, and femoral heads were recorded and compared to the original brachytherapy treatment plan for all 15 VMAT plans. In addition to the evaluation of the maximum dose points of the bladder and rectum, the amount of dose received by 5% of the bladder and rectal volumes were also recorded and compared to estimate the increase of integral dose when using VMAT treatment planning techniques.

The method of comparing isodose volumes was as follows: the amount of dose received by 99.0% of the dose volume structures in the VMAT plans were compared to the same 99.0% volume of the dose volumes in the brachytherapy plan. The dose volume structures were created by converting the 200%, 150%, 130%, 100%, 75%, and 50% dose volumes into structures using the “Convert Dose Volume to Structure” tool in the Eclipse TPS. To clarify, the 200% dose volume structure is equivalent to the volume receiving 12 Gy(6 Gy × 200%) from the original brachytherapy plan, the 150% dose volume structure is equivalent to the volume receiving 9 Gy(6 Gy × 150%) from the original brachytherapy plan, and so on. The previously mentioned dosimetric evaluation points and volumes of the five treatment plans in each type were averaged and then compared to those of the brachytherapy treatment plan.

## III. RESULTS & DISCUSSION

The dose received by 99% of the 200% dose volume structure was 11.96 Gy in the original brachytherapy treatment plan. The amount of dose received by 99% of the 200% dose volume structure in the Type 1 VMAT treatment plans was on average 11.76 Gy. The 150% dose volume structure received a dose of 9.01 Gy in the brachytherapy plan and on average 9.19 Gy in the Type 1 VMAT treatment plans to 99% of its volume. Ninety‐nine percent of the 130% dose volume structure received 7.82 Gy in the brachytherapy plan and on average 7.91 Gy in the Type 1 VMAT plans. Ninety‐nine percent of the 100% dose volume structure received 5.987 Gy in the brachytherapy plan and on average 5.967 Gy in the Type 1 VMAT plans. Ninety‐nine percent of the 75% dose volume structure received 4.53 Gy in the brachytherapy plan and on average 4.35 Gy in the Type 1 VMAT plans. Lastly, 99% of the 50% dose volume structure received 3.03 Gy in the brachytherapy plan and on average 2.84 Gy in the VMAT plans. It can be said that the dose coverage of the 200%–100% dose volumes of the brachytherapy and the Type 1 VMAT treatment plans agreed on average within 2%. Furthermore, the 75% dose volume coverage was on average 4.0% less than the BT plan, and the 50% dose volume coverage was on average 6.3% less than the BT plan (see Table 1).

The agreement among the higher isodose volumes is better than that of the lower isodose volumes. This is consistent with the previously published results of Malhotra et al.^(12)^ In the study completed by Malhotra and colleagues, a seven‐field intensity‐modulated radiotherapy treatment plan was utilized for the duplication of tandem and ovoid distributions. Excellent agreement with the original brachytherapy plan for isodose values of 75% or higher was observed. It was also observed that the agreement between the brachytherapy plan and the IMRT plan decreased when comparing isodose levels lower than 75%. According to Malhotra et al., the maximum dose point to the bladder and rectum increased on average 0.47 Gy and 2.34 Gy, respectively, in the Type 1 VMAT treatment plans. The dose to 5% of the bladder and rectum decreased on average 0.10 Gy and 0.36 Gy respectively in the Type 1 VMAT plans (see Table 2).

In our study, the Type 2 VMAT treatment plans were analyzed by comparing the isodose volumes, as well as dose to OARs. The main goal when creating the second set of VMAT plans was to ensure that the 100% dose volume structure received dose coverage comparable to that of the brachytherapy plan while delivering the lowest dose achievable to the OARs.

The dose to 99% of the 100% dose volume structure was on average 6.02 Gy for the Type 2 of the VMAT treatment plans. This is only a 0.51% increase of dose to the PTV when compared to the brachytherapy plan. Ninety‐nine percent of the 200%, 150%, and 130% dose volume structures received on average 6.88 Gy. The dose to 99% of the 75% and 50% dose volume structures was decreased 19.5% and 63.7% in comparison to the original brachytherapy plan (see Table 3). On average, the maximum dose point to the bladder was reduced by 0.15 Gy, and the dose to 5% of the bladder volume decreases on average 0.82 Gy in the Type 2 VMAT plans in comparison to the brachytherapy plans. The maximum dose point to the rectum increased on average 0.39 Gy, while the dose to 5% of the rectal volume decreased on average 1.37 Gy (Table 4).

The Type 3 VMAT treatment plans were analyzed by comparing the isodose volumes, as well as dose to OARs. The main goal when creating this third set of VMAT plans was to ensure that the 100% dose volume structure obtained an increased dose compared to that of the brachytherapy plan, all while delivering the lowest achievable dose to the OARs.

The dose to 99% of the 100% dose volume structure was on average 7.80 Gy in the Type 3 VMAT treatment plans, a 30.28% increase of dose to the PTV when compared to the brachytherapy plan. Ninety‐nine percent of the 200%, 150%, and 130% dose volume structures received on average 9.03 Gy. The dose to 99% of the 75% and 50% dose volume structures was increased by 0.90 Gy and decreased by 0.78 Gy in comparison to the original brachytherapy plan (Table 5). On average, the maximum dose point to the bladder increased by 1.12 Gy, and the dose to 5% of the bladder volume decreases on average 0.07 Gy in the Type 3 VMAT plans in comparison to the brachytherapy plans. The maximum dose point to the rectum increased on average 2.50 Gy and the dose to 5% of the rectal volume increased on average 0.44 Gy (Table 6).

**Table 1:** Isodose volume evaluation for Type 1 of the VMAT plans: evaluation of dose to 99% of the dose volume structures for the VMAT treatment plans created with the intent of duplicating the tandem and ovoid dose distribution to the original brachytherapy plan

*Dose Volume Structure*	*Brachytherapy Plan (Gy)*	*VMAT Plans Avg*. ±SD *(Gy)*
12 Gy‐200%Rx	11.96	11.76±0.11
9 Gy‐150%Rx	9.01	9.19±0.19
7.8 Gy‐130%Rx	7.82	7.91±0.22
6 Gy‐100%Rx	5.99	5.97±0.25
4.5 Gy‐75%Rx	4.53	4.35±0.23
3 Gy‐50%Rx	3.03	2.84±0.18

**Table 2:** Evaluation of dose points and dose to OARs for Type 1 of the VMAT plans showing a comparison of dose to points A and B, maximum dose points to the bladder, rectum and femoral heads, as well as the dose to 5% of the bladder and rectal volumes for set 1 of the VMAT plans to the original brachytherapy plan

*Dose Points and Volumes*	*Brachytherapy Plan (Gy)*	*VMAT Plans Avg*. ±SD *(Gy)*
Point A Rt.	5.68	6.88±0.37
Point A Lt.	5.99	6.60±0.27
Point B Rt.	1.74	3.39±0.30
Point B Lt.	1.74	3.33±0.23
Bladder	5.14	5.61±0.17
Rectum	5.15	7.48±0.31
Lt. Femoral Head	0.78	2.41±0.32
Rt. Femoral Head	1.03	2.37±0.12
5% Bladder Volume	3.94	3.84±0.19
5% Rectum Volume	5.49	5.13±0.23

**Table 3:** Isodose volume evaluation for Type 2 of the VMAT plans: evaluation of dose to 99% of the dose volume structures for the VMAT treatment plans created with the intent of delivering a homogeneous dose distribution of 6 Gy to the PTV to the original brachytherapy plan

*Dose Volume Structure*	*Brachytherapy Plan (Gy)*	*VMAT Plans Avg*. ±SD *(Gy)*
12 Gy‐200%Rx	11.96	6.89±0.53
9 Gy‐150%Rx	9.01	6.88±0.52
7.8 Gy‐130%Rx	7.82	6.87±0.51
6 Gy‐100%Rx	5.99	6.02±0.17
4.5 Gy‐75%Rx	4.53	3.65±0.07
3 Gy‐50%Rx	3.03	1.10±0.07

**Table 4:** Evaluation of dose points and dose to oars for Type 2 of the VMAT plans, showing comparison of dose to points A and B, maximum dose points to the bladder, rectum and femoral heads as well as the dose to 5% of the bladder and rectal volumes for set 2 of the VMAT plans to the original brachytherapy plan

*Dose Points and Volumes*	*Brachytherapy Plan (Gy)*	*VMAT Plans Avg* ±SD *(Gy)*
Point A Rt.	5.68	6.46±0.47
Point A Lt.	5.99	6.97±0.86
Point B Rt.	1.74	3.13±0.40
Point B Lt.	1.74	3.46±0.49
Bladder	5.14	4.99±0.41
Rectum	5.15	5.53±0.24
Lt. Femoral Head	0.78	2.50±0.38
Rt. Femoral Head	1.03	2.57±0.27
5% Bladder Volume	3.94	3.12±0.06
5% Rectum Volume	5.49	4.12±0.28

The dose to point A and point B increased in all 15 of the VMAT treatment plans with a maximum average increase in dose of 3.58 Gy to point A in the Type 3 VMAT plans. This is to be expected since the dose regimen of the third set of VMAT plans was altered to deliver a larger dose to the PTV and, in turn, a larger dose to point A. The dose to the femoral heads increased in all of the VMAT treatment plans, with a maximum increase of 2.95 Gy on average in Type 3 of the VMAT plans. Attempting to increase the dose to the PTV with a homogeneous dose distribution resulted in an increase in maximum dose and dose volumes of OARs. The dosimetrically best plans from the Type 3 VMAT plans were those that utilized multiple isocenters for the multiple full/partial arcs.

Multiple studies of intensity‐modulated radiation therapy for the treatment of gynecologic malignancies have previously been done. It was noted in previous studies that in comparison to three‐dimensional conformal radiation therapy, the irradiated volumes of critical structures decreased. Also the irradiated volumes of critical structures decreased with increasing number of fields when using IMRT planning techniques.^13^, ^14^, ^15^, ^16^, ^17^ Since VMAT is essentially multiple IMRT fields continuously delivered over an arc, this finding led to the motivation behind using VMAT to potentially deliver a more conformal dose to the PTV and decreased dose to organs at risk.^(18)^

**Table 5:** Isodose volume evaluation for Type 3 of the VMAT plans: evaluation of dose to 99% of the dose volume structures for the VMAT treatment plans created with the intent of delivering an increased homogeneous dose distribution to the PTV to the original brachytherapy plan

*Dose Volume Structure*	*Brachytherapy Plan (Gy)*	*VMAT Plans Avg* ±SD *(Gy)*
12 Gy‐200%Rx	11.96	9.25±0.19
9 Gy‐150%Rx	9.01	9.03±0.19
7.8 Gy‐130%Rx	7.82	8.81±0.15
6 Gy‐100%Rx	5.99	7.80±0.20
4.5 Gy‐75%Rx	4.53	5.43±0.26
3 Gy‐50%Rx	3.03	2.25±0.24

**Table 6:** Evaluation of dose points and dose to OARs for Type 3 of the VMAT plans showing Comparison of the dose to points A and B, maximum dose points to the bladder, rectum and femoral heads as well as the dose to 5% of the bladder and rectal volumes for set 3 of the VMAT plans to the original brachytherapy plan

*Dose Points and Volumes*	*Brachytherapy Plan (Gy)*	*VMAT Plans Avg* ±SD *(Gy)*
Point A Rt.	5.68	9.26±0.59
Point A Lt.	5.99	8.42±0.21
Point B Rt.	1.74	4.52±0.43
Point B Lt.	1.74	4.37±0.34
Bladder	5.14	6.26±0.24
Rectum	5.15	7.64±0.66
Lt. Femoral Head	0.78	3.81±0.42
Rt. Femoral Head	1.03	3.98±0.50
5% Bladder Volume	3.94	3.87±0.34
5% Rectum Volume	5.49	5.93±0.27

Toxicity to organs at risk should always be considered when using any external beam radiation therapy technique to treat those who are unable to receive brachytherapy treatment. It is possible for patients to develop high‐grade toxicity to their gastrointestinal tracks as severe as grade 3 rectal bleeding.^(18)^ Several studies have examined the recurrence of the gynecologic malignancies when using external beam stereotactic body radiation therapy dose regimens in lieu of brachytherapy for gynecologic cases, and have reported significant toxicity including grade 4 enterovaginal fistulas and grade 4 ileus.^19^, ^20^, ^21^ Toxicity is still a great risk and should be conservatively addressed when planning these clinically challenging cases.

## IV. CONCLUSIONS

Brachytherapy provides patients with gynecologic malignancies a highly conformal dose to treatment volumes and limits irradiation of healthy tissue to an acceptable level. However, not all patients are able or willing to receive such treatment and require alternative methods for controlling the progression of their disease. VMAT shows great potential for producing highly conformal doses to treatment volumes while sparing OARs. VMAT was successful in duplicating the high‐dose volumes (200%–75%) of the brachytherapy treatment plans, as well as delivering a homogenous dose distribution of 6 Gy to the PTV. VMAT was unable to deliver an increased homogenous dose to the PTV while appropriately sparing the OARs. VMAT could potentially be an alternative option for duplicating traditional brachytherapy dose distributions for patients in need of brachytherapy who are unable to undergo the treatment modality. Toxicity to critical structures is still a great risk and should be conservatively addressed, along with patient motion management and intrafractional tumor regression, when planning these clinically challenging cases.

## Supporting information

Supplementary Material

Supplementary Material

Supplementary Material

Supplementary Material

Supplementary Material

Supplementary Material

## References

[1] Thomadsen BR , Lin SW , Laemmrich P et al. Analysis of treatment delivery error in brachytherapy using formal risk analysis techniques. Int J Radiat Oncol Biol Phys. 2003;57(5):1492–508. 14630289 10.1016/s0360-3016(03)01622-5

[2] National Comprehensive Cancer Network. NCCN guidelines: cervical cancer, version 1.2012. Fort Washington, PA: NCCN; 2010.

[3] Chan P , Yeo I , Perkins G , Fyles A , Milosevic M . Dosimetric comparison of intensity‐modulated, conformal, and four‐field pelvic radiotherapy boost plans for gynecologic cancer: a retrospective planning study. Radiat Oncol. 2006;1:13. 16722546 10.1186/1748-717X-1-13 PMC1471795

[4] Barraclough LH , Swindell R , Livsey JE , Hunter RD , Davidson SE . External beam boost for cancer of the cervix uteri when intracavitary therapy cannot be performed. Int J Radiat Oncol Biol Phys. 2008;71(3):772–78. 18207658 10.1016/j.ijrobp.2007.10.066

[5] Mundt AJ , Lujan AE , Rotmensch J et al. Inensity‐modulated whole pelvic radiotherapy in women with gynecologic malignancies. Int J Radiat Oncol Biol Phys. 2002;52(5):1330–37. 11955746 10.1016/s0360-3016(01)02785-7

[6] Kemmerer E , Hernandez E , Ferriss J et al. Use of image‐guided stereotactic body radiation therapy in lieu of intracavitary brachytherapy for the treatment of inoperable endometrial neoplasia. Int J Radiat Oncol Biol Phys. 2013;85(1):129–35. 22503523 10.1016/j.ijrobp.2012.02.058

[7] Grimm J , LaCouture T , Croce R , Yeo I , Zhu Y , Xue J . Dose tolerance limits and dose volume histogram evaluation for stereotactic body radiation therapy. J Appl Clin Med Phys. 2011;12(2):267–92. 10.1120/jacmp.v12i2.3368 PMC5718687 21587185

[8] Timmerman RD . An overview of hypofractionation and introduction to this issue of seminars in radiation oncology. Semin Radiat Oncol. 2008;18(4):215–22. 18725106 10.1016/j.semradonc.2008.04.001

[9] Khan FM . The physics of radiation therapy. 4th edition Baltimore, MD: Kluwer/Lippincott Williams & Wilkins; 2010.

[10] Kwon JS , Stuhr K , Winter K , Schefter T , Dicker AP . RTOG 0417. A phase II study of bevacizumab in combination with definitive radiotherapy and cisplatin chemotherapy in untreated patients with locally advanced cervical carcinoma. Radiation Therapy Oncology Group 0417:1–85; 2011.

[11] Podgorsak EB . Radiation oncology physics: a handbook for teachers and students, 1st edition Vienna, Austria: IAEA; 2005.

[12] Malhotra HK , Avadhani JS , de Boer SF , Jaggernauth W , Kuettel MR , Podgorsak MB . Duplicating a tandem and ovoids distribution with intensity‐modulated radiotherapy: a feasibility study. J Appl Clin Med Phys. 2007;8(3):91–98. 10.1120/jacmp.v8i3.2450 PMC5722607 17712303

[13] Roeske JC , Lujan A , Rotmensch J , Waggoner SE , Yamada D , Mundt AJ . Intensity‐modulated whole pelvis radiation therapy in patients with gynecologic malignancies. Int J Radiat Oncol Biol Phys. 2000;48(5):1613–21. 11121668 10.1016/s0360-3016(00)00771-9

[14] Mundt AJ , Roeske JC , Lujan AE . Intensity‐modulated radiation therapy in gynecologic malignancies. Med Dosim. 2002;27(2):131–36. 12074464 10.1016/s0958-3947(02)00095-x

[15] Heron DE , Gerszten K , Selvaraj RN et al. Conventional 3D conformal versus intensity‐modulated radiotherapy for the adjuvant treatment of gynecologic malignancies: a comparative dosimetric study of dose‐volume histograms. Gynecol Oncol. 2003;91(1):39–45. 14529660 10.1016/s0090-8258(03)00461-x

[16] Ahamad A , D'Souza W , Salehpour M et al. Intensity modulated radiation therapy after hysterectomy: comparison with conventional treatment and sensitivity of the normal‐tissue‐sparing effect to margin size. Int J Radiat Oncol Biol Phys. 2005;62(4):1117–24. 15990017 10.1016/j.ijrobp.2004.12.029

[17] Wong E , D'Souza DP , Chen JZ et al. Intensity‐modulated arc therapy for treatment of high‐risk endometrial malignancies. Int J Radiat Oncol Biol Phys. 2005;61(3):830–41. 15708263 10.1016/j.ijrobp.2004.06.253

[18] Otto K . Volumetric modulated arc therapy: IMRT in a single gantry arc. Med Phys. 2008;35(1):310–16. 18293586 10.1118/1.2818738

[19] Higginson DS , Morris DE , Jones EL , Clarke‐Pearson D , Varia MA . Stereotactic body radiotherapy (SBRT): technological innovation and application in gynecologic oncology. Gynecol Oncol. 2001;120(3):404–12. 10.1016/j.ygyno.2010.11.042 21194733

[20] Hsieh CH , Wei MC , Hsu YP et al. Should helical tomotherapy replace brachytherapy for cervical cancer? Case report. BMC Cancer. 2010;10:637. 21092235 10.1186/1471-2407-10-637 PMC3001446

[21] Guckenberger M , Bachmann J , Wulf J et al. Stereotactic body radiotherapy for local boost irradiation in unfavourable locally recurrent gynaecological cancer. Radiother Oncol. 2010;94(1):53–59. 20079550 10.1016/j.radonc.2009.12.004

