# Supplementary figures and images for: VMAT for the treatment of gynecologic malignancies for patients unable to receive HDR brachytherapy
Source: J Appl Clin Med Phys. 2014 Sep 8;15(5):66–73. doi: 10.1120/jacmp.v15i5.4839 (PMC5711077; doi:10.1120/jacmp.v15i5.4839)

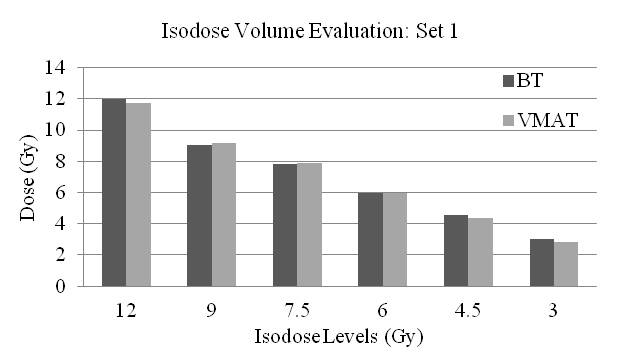

Supplement: Supplementary file 1 — Supplementary Material [file ACM2-15-066-s001.jpg]

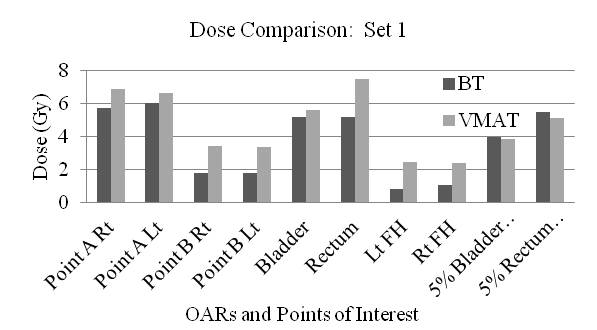

Supplement: Supplementary file 2 — Supplementary Material [file ACM2-15-066-s002.jpg]

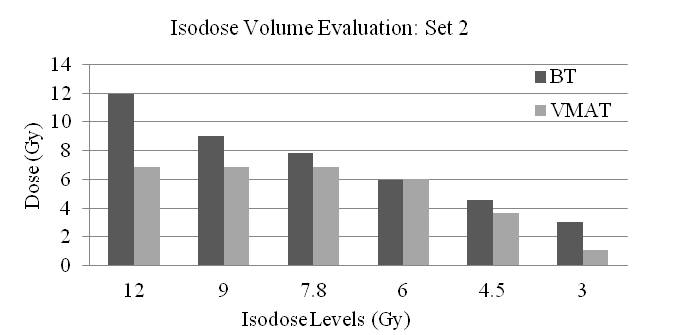

Supplement: Supplementary file 3 — Supplementary Material [file ACM2-15-066-s003.jpg]

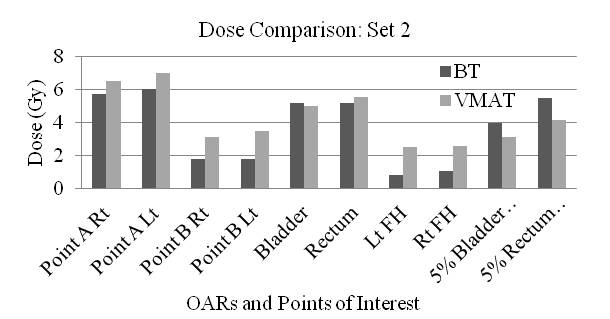

Supplement: Supplementary file 4 — Supplementary Material [file ACM2-15-066-s004.jpg]

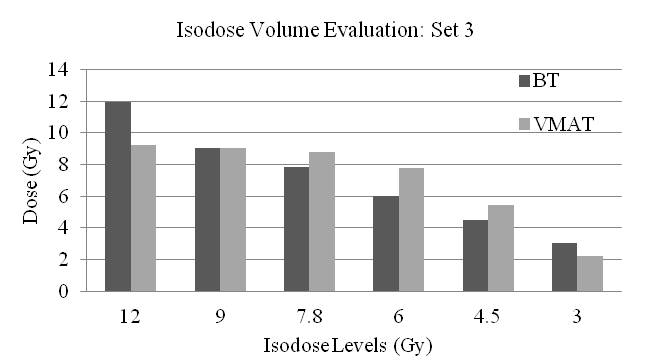

Supplement: Supplementary file 5 — Supplementary Material [file ACM2-15-066-s005.jpg]

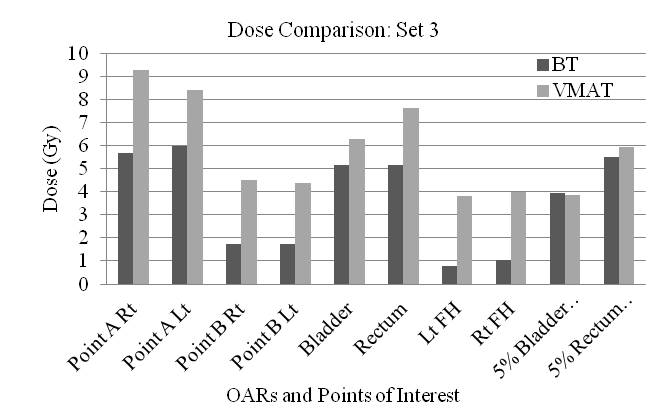

Supplement: Supplementary file 6 — Supplementary Material [file ACM2-15-066-s006.jpg]
